# Test-retest reliability of the “Home Town Walk” fMRI paradigm for memory activation and lateralization in the pre-surgical evaluation of patients with temporal lobe epilepsy

**DOI:** 10.3389/fneur.2024.1419047

**Published:** 2024-07-23

**Authors:** Rosa M. Sanchez Panchuelo, Robert Flintham, Roman Wesolowski, Roya Jalali, Jane Herbert, Shanika Samarasekera, Andrew P. Bagshaw, Ramesh Chelvarajah, Nigel P. Davies, Vijay Sawlani

**Affiliations:** ^1^Medical Physics, University Hospitals Birmingham NHS Foundation Trust, Birmingham, United Kingdom; ^2^Imaging, University Hospitals Birmingham NHS Foundation Trust, Birmingham, United Kingdom; ^3^Complex Epilepsy Surgery, University Hospitals Birmingham NHS Foundation Trust, Birmingham, United Kingdom; ^4^Centre for Human Brain Health and School of Psychology, University of Birmingham, Birmingham, United Kingdom

**Keywords:** fMRI, memory, epilepsy, Home Town Walk, reproducibility

## Abstract

**Introduction:**

Functional magnetic resonance imaging (fMRI) can be used to assess language and memory function as part of pre-surgical decision making in refractory epilepsy. Although language paradigms are well established, memory paradigms are not widely used in clinical practice due to a lack of evidence for robust and reliable methods. Here, we aim to investigate the clinical utility of the Home Town Walk (HTW) paradigm for personalized treatment decisions in medial temporal lobe epilepsy.

**Methods:**

A cohort of 123 consecutive patients having HTW-fMRI as part of routine MRI scans over a 7.5 year period were included in this retrospective study. Of these, 111 patients underwent repeated HTW-fMRI in two scanning sessions one to three days apart. fMRI analysis was performed at the time of the scans using clinically approved software and retrospectively validated using FSL. We assessed the test–retest within subject reliability of activations within the posterior parahippocampal gyri (pPHG) at the individual subject level.

**Results and discussion:**

Activations within the pPHG region were observed for 101 patients (91%) in at least one of the fMRI sessions and for 88 patients (79%) in both fMRI sessions, with 82 patients showing overlapping unilateral or bilateral activations and 8 further patients showing overlapping activations in one of the hemispheres but not the other. Reproducibility was evaluated using metrics based on the concordance ratios for size (R_size_) and location (R_overlap_) within the pPHG region, as well as the lateralization index (LI) metric to reflect the asymmetry of hemispheric activations, which is of crucial relevance to inform surgery. Test–retest reliability of visuospatial memory LIs, assessed by an intra-class correlation coefficient (ICC) yielded a value of 0.76, indicating excellent between session stability of memory lateralization.

**Conclusion:**

The HTW-fMRI paradigm shows reproducible activations in the medial temporal lobes of individual epilepsy patients sufficient to consistently lateralize visuospatial memory function, demonstrating the clinical utility of HTW memory fMRI and its potential for application in the pre-surgical assessment of people with temporal lobe epilepsy.

## 1 Introduction

Selection of patients for surgical management of intractable temporal lobe epilepsy (TLE) is a complex clinical pathway, including seizure activity assessment, electroencephalogram (EEG), video telemetry, structural MRI, positron emission tomography (PET), and mapping of eloquent brain functions (1). Concordance in the localisation of seizures electrographically, radiographically, and semiologically is associated with good surgical results in terms of seizure freedom. However, post-operative memory decline remains a significant complication after surgery, and it has been shown to correlate with the side of surgical intervention (2–4).

The medial temporal lobe (MTL) has a specific role in episodic memory encoding and retrieval (5), with the involvement of key anatomical regions such as the hippocampus, parahippocampal gyrus (PHG) and amygdala in memory processing. Surgery involving these MTL structures can therefore have negative effects on memory (6). Additionally, memory impairment is a common cognitive comorbidity in TLE patients (7, 8). Patients undergoing resection surgery of the left anterior temporal lobe are typically at risk for verbal memory decline, whereas patients undergoing resection surgery of their right anterior temporal lobe are most at risk of visual memory loss (8, 9).

Recent developments in neuroimaging, particularly in the field of fMRI, have facilitated the non-invasive identification of eloquent cortex and the localisation and lateralization of language and memory functions. Some studies on preoperative memory using functional Magnetic Resonance Imaging (fMRI) have shown that individual patients with relatively greater activation in the temporal lobe region intended for resection (ipsilateral) compared with contralateral medial temporal lobe activation had greater memory decline following anterior temporal lobe resection (ATLR) (4, 10, 11), and this was the case for both verbal memory decline following dominant ATLR and for non-verbal memory decline following non-dominant ATLR (11). Conversely, if the contralateral (non-operated) hemisphere shows significant compensatory activity, the risk of memory decline may be lower. Therefore, pre-surgical evaluation of the lateralization and localization of memory and language is vital to predict the risk of significant post-operative memory and language deficits. The American Academy of Neurology (AAN) practice guidelines on the use of fMRI for presurgical mapping in epilepsy (12) suggest that fMRI may be considered as an alternative option to the intracarotid amobarbital procedure (Wada Test – WT) for lateralizing language and memory functions in patients with MTL epilepsy. Crucially, these guidelines suggest that fMRI of verbal memory or language encoding should be considered for predicting verbal memory outcome, while fMRI using nonverbal memory encoding may be considered for predicting visuospatial memory outcomes in patients undergoing presurgical evaluation for possible temporal lobectomy. Although WT is still indicated in patients at risk for developing global amnesia (those with significant bilateral or contralateral memory deficits), WT has a limited role in predicting memory functions after right sided ATLR (13) and was also insufficiently reliable to accurately localize verbal memory processes after left sided ATLR surgery (14). In a review that compares the current role of WT and fMRI in the presurgical evaluation of TLE patients, WT exhibited no added value, beyond preclinical data, for predicting material-specific memory impairment, whereas fMRI was reliable for either verbal or non-verbal memory decline (15). As a result, and given the invasiveness of WT, fMRI has progressively replaced WT in many hospitals (16, 17).

In contrast to language fMRI, where paradigms are well established (18), memory paradigms have been difficult to use clinically. Most fMRI studies in MTLE have been performed at the group level (2, 4, 10, 11, 19–21), although Sidhu et al. (21) showed that left lateralization within frontal and anterior medial temporal activations evoked with a verbal memory encoding task could predict verbal memory post-surgery outcome in individual patients. However, obtaining memory specific robust activations at the individual level has proven challenging with mixed results across fMRI studies (22, 23). The difficulty is to design paradigms that can evoke robust enough BOLD responses within the MTL region at the individual level, particularly in patients where memory processes are impaired. There is no consensus on the optimal memory paradigm due to the wide range of aspects that need to be considered such as the specific memory process that should be targeted, or the cognitive ability of patients and how it might influence task performance (24, 25). Although studies assessing the test–retest reproducibility of memory specific activations in individual patients are scarce, the available data suggest that most memory paradigms have not provided reproducible brain activations in epilepsy patients in the clinical setting (26–28), restricting the clinical utilization of memory fMRI. One study (29) investigated the between session reliability of MTL activations for a series of memory paradigms which had demonstrated predictive value for postoperative memory outcome in a small number of epilepsy patients, and found fair to good reliability with Home Town Walk and scene encoding paradigms but low reliability with picture encoding and word encoding paradigms.

The Home Town Walk (HTW) paradigm (30) is predominantly a visuospatial memory retrieval task where participants mentally navigate a familiar walk, for example through their home town. This mental navigation task may also involve verbal strategies and other imagined stimuli in addition to spatial navigation strategies. It has been shown that this paradigm can evoke strong activation in the MTL region (4, 29) and can predict post-operative visual memory loss after anterior temporal lobectomy in right-sided medial temporal lobe epilepsy (MTLE) (4). Given the relatively superior between sessions reproducibility of medial temporal lobe activations produced by the HTW compared to other memory paradigms shown by Towgood et al. (29), and own experience of this task producing more robust activations compared to other memory tasks, we chose to use this paradigm to lateralize visual memory function in our clinical practice. This paradigm is used in conjunction with several language fMRI paradigms, including language encoding, which has been recommended as an option for predicting verbal memory outcome in the AAN guidelines (12). By combining information from all language and memory fMRI assessments, we aim to provide more accurate and personalized information for surgical planning.

The primary objective of our study was to demonstrate the effectiveness of the HTW fMRI paradigm in routine clinical practice for reproducibly localizing visuospatial memory retrieval in focal epilepsy patients undergoing presurgical evaluation. In particular, given that previous fMRI memory studies have found that the relative distribution of left and right activation is a useful measure to predict post-surgical memory outcome, we assess the reliability of memory hemispheric lateralization or asymmetry index (LI). The (LI) metric was generated based on the relative ratio of activated voxels across hemispheres within automated regions defined by the posterior parahipocampal gyrus (PHG) probabilistic atlas. By assessing the robustness and reproducibility of the HTW paradigm in a large patient cohort at the individual patient level using both clinically available and state-of-the-art fMRI analysis tools, we aimed to validate its clinical usefulness and establish its reliability and potential for guiding surgical interventions to minimize the risk of post-operative memory deficits.

Note that since most patients in our study have not undergone surgery, an assessment of the HTW paradigm to predict postoperative memory loss is beyond of the scope of this study and this will be assessed in future work.

## 2 Materials and methods

### 2.1 Patient cohort

**Figure 1:**
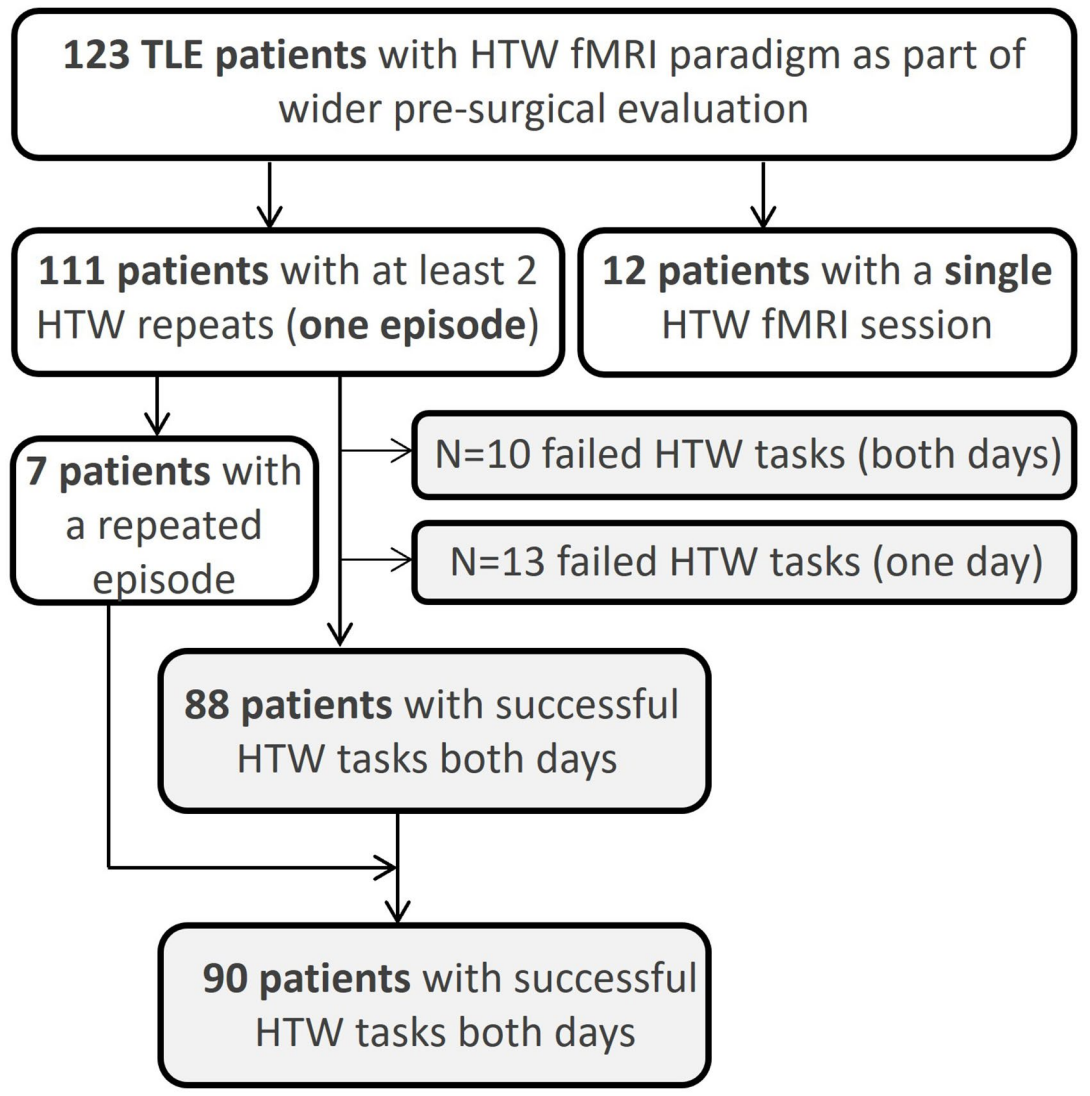
Overview of number of patients scanned with the HTW fMRI paradigm. Reproducibility metrics were assed across patients where the HTW successfully evoked significant BOLD activations within the pPHG ROI across two fMRI sessions.

Data were collected at a major neurosurgical center between June 2015 and Dec 2022 on patients with epilepsy being assessed by the Epilepsy Surgery Service. Patients underwent fMRI using a block-design HTW memory paradigm as part of a routine clinical MRI protocol including language fMRI assessment. The language paradigms typically consist of silent Word Generation, Noun-Verb Association and Sentence Completion. In patients with cognitive or literacy impairment we employ a Picture Recognition paradigm whereas for patients with visual impairment, audio-based paradigms such as Story Listening are used. These additional paradigms are decided on a patient-by-patient basis. For reproducibility assessment, all patients were invited for a repeat fMRI session one to three days after the first session. For the purpose of the analysis, two HTW-fMRI datasets within three days were considered a single complete episode of memory fMRI. A total of 248 HTW-fMRI datasets from 123 patients (67 males, age: 38 ± 12 years, ranging from 18 to 70 years old) were assessed (Figure 1). This retrospective analysis included all patients identified as having undergone HTW fMRI in the time period covered. One hundred and eleven patients had at least one complete memory fMRI episode; five of these patients had a total of three fMRI sessions on different days. This is because it was clear they had not prepared a HTW prior to the first session, resulting in poor performance of the task. In these cases, where patients were recalled to complete a new episode after the first day, the last two sessions were evaluated for reproducibility. Seven of the 111 patients had a complete second episode of memory fMRI (a total of 4 fMRI scan sessions).

Note that patient details relating to duration and severity of epilepsy, presence of lesions or neuropsychologic evaluation assessing memory impairment are not included as analysis of these in relation to the fMRI results is beyond the scope of this paper. Our goal is to assess reproducibility at the individual patient level across a large cohort of patients with variable disease characteristics and degrees of memory impairment.

### 2.2 Paradigm

The HTW paradigm requires mental navigation through one’s home town by using landmarks determined by the patients themselves. Patients were instructed to prepare their familiar walk prior to the first session (the patient information sheet for the HTW is provided as Supplementary material). Patients were instructed to start and end at familiar places, divide the route into 10 segments, with each waypoint as the starting point for the next segment. The fMRI HTW paradigm consisted of a block design comprising the 10 prepared ‘Walk’ segments alternating with ‘Control’ block periods, each of 30 s duration, with the total task lasting 10 min. During the fMRI scans, patients were cued on the screen to visualize and recount in their heads each segment of their prepared detailed walk to the best of their abilities during the ‘Walk’ periods, and to silently count down from 100 during the ‘Control’ periods. Each participant used the same prepared walk for both sessions.

### 2.3 MRI protocol

MRI data were collected across two MRI scanners: Siemens MAGNETOM Verio 3 T (Scanner 1) and Siemens MAGNETOM Skyra 3 T (Scanner 2) (Siemens, Erlangen, Germany) using a head coil with 32-receive channels. Functional MRI data were acquired in axial orientation using T2∗-weighted, multi-slice, single-shot gradient echo–echo planar imaging (GE-EPI) with parameters shown in Table 1. A structural T1-weighted volume was acquired in axial orientation using MPRAGE (0.65 × 0.65 × 1 mm^3^ resolution, 176 slices, TR_MPRAGE_ = 1700 ms, TI = 900 ms, TE = 2.91 ms, FA = 9°). Note that two of the patients with repeated episodes were scanned using Scanner 1 for the first episode and Scanner 2 for the second episode.

**Table 1:** Details of the EPI acquisition protocol for each scanner.

	Scanner 1	Scanner 2
FOV	250×250 mm^2^	250×250 mm^2^
Acquisition matrix	64 × 64	64 × 64
Number of slices	36	35
Slice thickness	3.75 mm (25% gap)	4 mm (25% gap)
GRAPPA acceleration	2	2
TE	30 ms	30 ms
TR	3 s	2 s
Number of volumes	200	300

### 2.4 Clinical analysis

**Figure 2:**
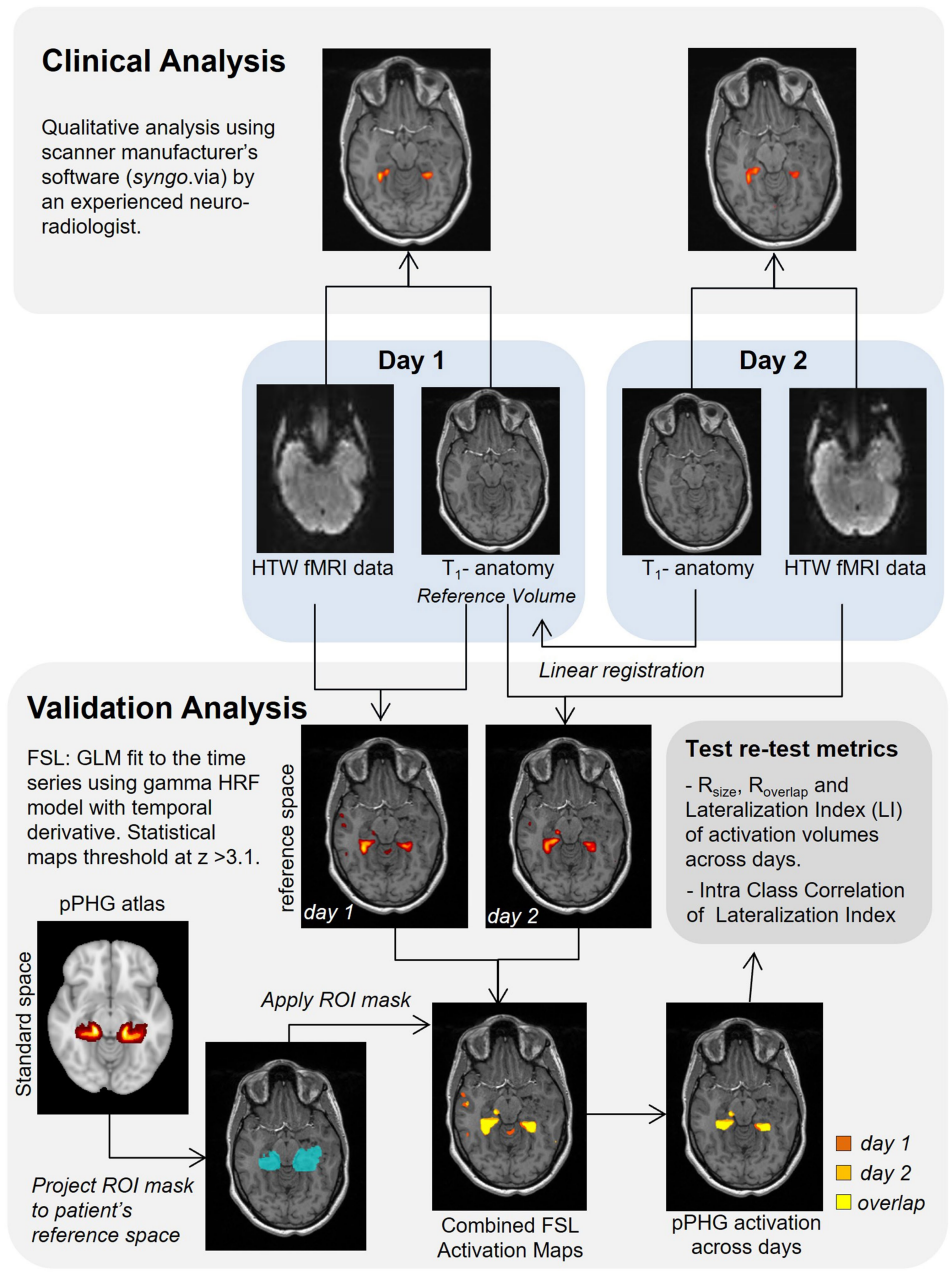
Schematic illustration of the analysis steps.

A diagram of the analysis steps is shown in Figure 2. The HTW-fMRI datasets from each individual patient were first qualitatively evaluated in the clinical setting by a single experienced Consultant Neuroradiologist (author *VS*). Data were analyzed using a General Linear Model (GLM) with the Neuro3D application in the manufacturer’s clinical image analysis software package “syngo.via” (Siemens, Erlangen, Germany). Threshold (t typically 3.9 or higher) and minimum cluster size of the t-statistical maps were subjectively varied to optimize a perceived balance between pPHG activation and spurious signal for each patient. Clinical reports qualitatively evaluating HTW memory fMRI scans on the presence, lateralization and reliability of pPHG memory-specific activation across sessions were produced for surgical planning. Activations from each fMRI session were reviewed by MR physicists to identify the reported activation pattern as bilateral or predominantly left or right sided. Patients were then classified according to the reproducibility of the pPHG activation patterns observed across an episode; (A) Activation pattern from day 1 reproduced on day 2 (including lateralization), (B) different pPHG activation pattern on day 1 and day 2 (e.g., due to lack of activation in one of the days), and (C) No reliable pPHG activation on either day 1 or day 2.

### 2.5 Validation analysis

A second analysis to validate clinical results obtained as a part of the patient’s routine clinical care and quantify reproducibility of functional activation maps evoked with the HTW paradigm across sessions (days) was carried out using FMRIB Software Library, FSL tools (31). Pre-processing steps comprised realignment (motion correction) of all functional data to the middle volume of the functional data set (reference EPI volume), spatial smoothing using SUSAN noise reduction algorithm (the spatial extent of the smoothing determined by a Gaussian kernel of 5 mm at full width half maximum), temporal high-pass filtering (0.01 Hz cut-off) to account for scanner drift and other low-frequency signals and final time series were converted to percent-signal change for subsequent statistical analysis. Data were analyzed with FSL’s FEAT using a GLM where ‘Walk’ periods were modeled as a 30 s boxcar convolved with a canonical HRF model and its orthogonalized temporal derivative. The GLM was fitted using ordinary least-squares and the resulting statistical maps were created with a threshold at z-score > 3.1 (this corresponds to an uncorrected *p*-value threshold of 0.001, in line with a previous memory fMRI study (32)) and clustered using a p-cluster threshold of 0.05.

The statistical clustered z-stat activation maps from both days were transformed from native functional data acquisition space into the patient’s reference T1-weighted anatomical volume using FSL’s Linear Image Registration Tool (33–35). For patients with repeated scans, the T1-weighted (MPRAGE) anatomical volume collected on day 1 was used as patient’s reference anatomical volume, unless these data were missing in which case the anatomical volume collected on day 2 was used. For each day the reference EPI volume was aligned to the within-session T1-weighted volume in two steps; first, an initial transformation matrix was computed using FLIRT with the -useqform option, this matrix was then used as initial transform with FSL’s ‘epireg’ command to compute the final transform from EPI space to the within-session anatomy (for day one this was the transform to the reference anatomy). For day 2, a linear registration was computed between the anatomical volume from day 2 and the reference volume; this matrix was concatenated with the previous transformation matrix from EPI space to the within-session anatomy to compute the transform from EPI space to reference anatomy. Statistical maps were then registered to the reference anatomical space with a single transform matrix.

#### 2.5.1 Global BOLD evoked response to HTW paradigm

To provide an overview of the level of spatial overlap within each region of the cortex across all patients scanned, HTW activation maps for each patient were combined into standard space (MNI152 1 mm, see section 2.5.2 below for registration details) by adding binary masks of the activation map for each patient into standard space after non-linear registration. Only one HTW activation mask (from one of the days) was included for patients who underwent multiple fMRI HTW sessions. The combined map is hence a probabilistic atlas of HTW memory evoked BOLD responses based on the individual activation maps of the 123 patients scanned.

#### 2.5.2 Projection of pPHG atlas to patient’s reference space

Probabilistic masks of the posterior pPHG were used to provide quantitative HTW memory task ROI measures and to assess reproducibility across days. Masks of pPHG (right and left) regions were created from the FSL implementation of the Harvard-Oxford cortical atlas^1^ in standard MNI-152 (36) space (1 mm isotropic). These ROI masks were projected to each patient’s reference space for subsequent analysis.

The transform between the patient’s reference anatomical volume and the standard MNI space was computed using a two-pass process. The first pass involved a linear registration of the extracted brain from the reference anatomical volume to the MNI brain using FLIRT and then the resultant transformation was used as an initialization step for a non-linear transformation using FSL’s FNIRT. The resultant warp field was applied to project individual activation maps from each subject to standard MNI space (see section 2.4). FSL’s invwarp command was applied to compute warp fields from MNI to patient’s space, and project each of the probabilistic ROIs to each patient’s reference space.

#### 2.5.3 Within subject reproducibility of fMRI activation patterns across days

Patients were re-classified according to the reproducibility of the pPHG activation patterns based on the FSL analysis results into three groups as follows: (A) Activation patterns were deemed reproduced when there was overlap of activation volumes across days (see reproducibility metrics below) Activations were considered partially reproduced if there was overlap of activation within pPHG in one of the hemispheres (due to unilateral activation pattern in one day and bilateral activation pattern on the other). (B) Different activation patterns across days. (C) No significant pPHG activation on either day 1 or day 2.

**Figure 3:**
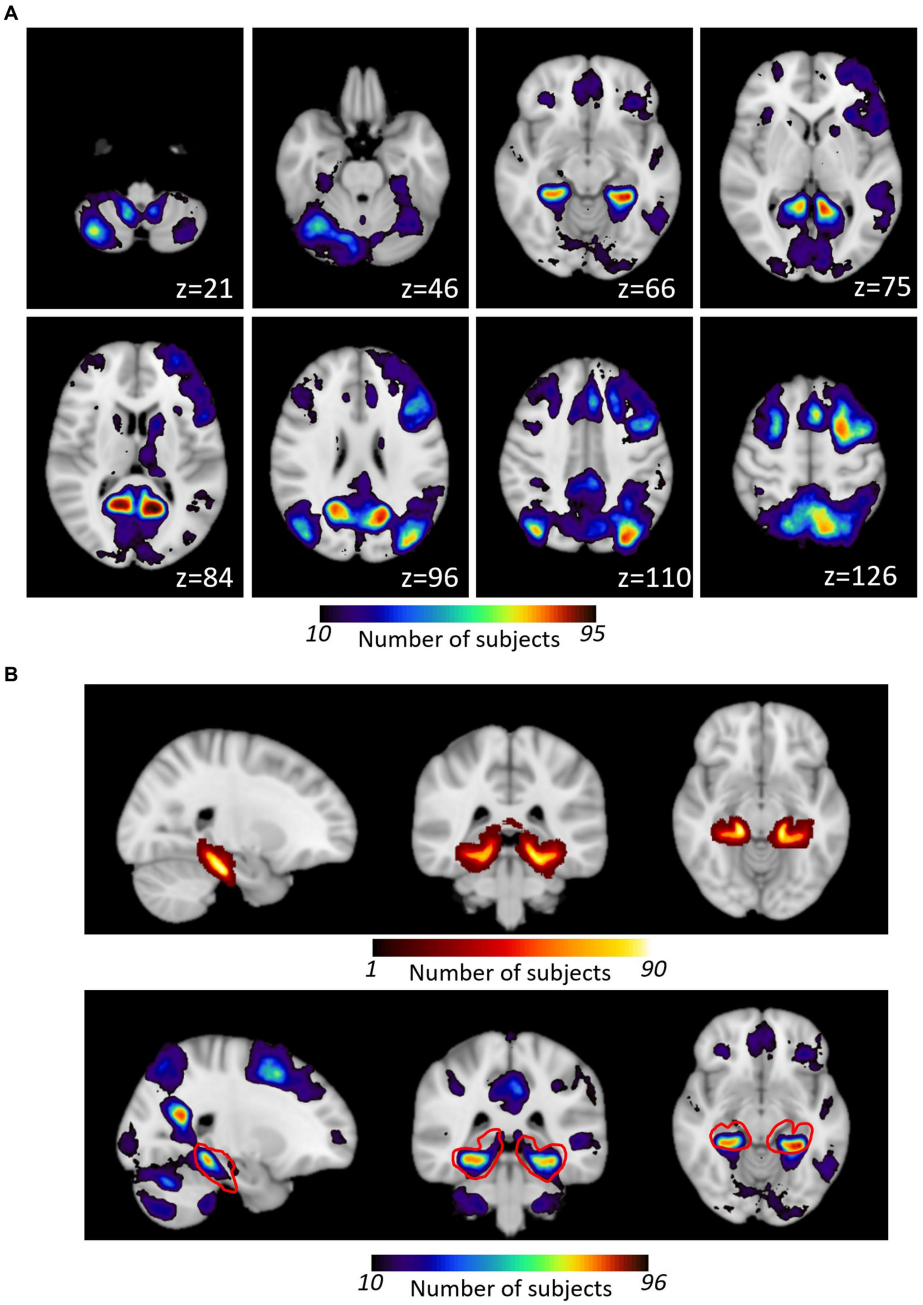
**(A)** Overlap of individual patient activation maps generated with the HTW memory task. Individual TLE patients’ maps were binarized and projected onto standard MNI152 template prior to be combined into a probabilistic map of HTW paradigm evoked activation. The color scale indicates the number of patients who activated that particular voxel. Axial slices displayed for a subset of slices (MNI z coordinate shown). **(B)** Comparison of Harvard Oxford pPHG probabilistic atlas (top) with HTW activation within that region (shown as red outline in bottom panel).

The degree of reproducibility of HTW evoked BOLD responses for patients who exhibited significant activations on both days within at least one of the pPHG hemispheric ROIs was evaluated by computing the following reproducibility measures. Reproducibility of the size of activated regions in the first and second days was assessed using the R_size_ metric (37):


$$R_{\mathrm{size}}=2\;\frac{V_{\text{min}}}{V_{1}+V_{2}}$$

where V_1_ and V_2_ are the activation volumes within the given ROI for the first and second days respectively, and V_min_ is the smallest of these volumes. To assess how well activations overlapped across days within each region, the relative amount of overlapping volume, R_overlap_, was computed according to the method proposed by Rombouts et al. (37):


$$R_{\mathrm{overlap}}=\frac{2\;V_{1}\cap V_{2}}{V_{1}+V_{2}}$$

where V_1_∩ V_2_ represents the intersection (or overlap) of activation volumes across days. The overlap metric can range from 0 to 1 and is based strictly on the location of significantly activated voxels. All volumes were calculated in the patient’s reference anatomical space based on binary activation masks generated for each day based on the statistical clustered z-stat activation maps and the probabilistic pPHG ROI (Figure 3).

In addition, the test–retest reproducibility of the hemispheric lateralization in the pPHG was assessed based on the lateralization index (LI). Lateralization indices were computed for each day based on the ratio of activation volumes within left and right pPHG ROIs:


$$LI=\frac{V_{\mathrm{left}}-V_{\mathrm{right}}}{V_{\mathrm{left}}+V_{\mathrm{right}}}$$

The LIs ranged from 100 (absolutely left lateralized) to −100 (absolutely right lateralized) and were classified as right lateralized if smaller than −20, left lateralized when greater than 20, and bilateral when LIs were in between (−20 ≤ LI ≤ 0) (38, 39). The consistency of the lateralization index was evaluated by examining the correlation (Pearson r) and the Intra-Class Correlation (ICC) based on a 2-way ANOVA analysis for LI across days performed using Microsoft Excel. The ICC represents the ratio of between-subject variance and between-tests variance; ICC approaches 1 when individual variability (across days) is smaller than the variability across individuals.

## 3 Results

Activation maps from all patients were combined in standard MNI152 space to form a probabilistic map of the BOLD responses evoked by the HTW paradigm. Nine of the 123 patients did not show any BOLD evoked responses to the HTW paradigm across the whole brain in either of the fMRI sessions (three of these patients were only scanned once), hence a total of 114 patients contributed to the combined map shown in Figure 3A (only one activation map per patient was included by selecting the session with the most significant BOLD responses in patients with more than one HTW fMRI session). This map shows the probability (i.e., number of patients) for each voxel in MNI standard space being activated by the HTW paradigm. BOLD activations were typically observed in the pre-cuneus cortex, the PHG and fusiform gyri and lateral occipital cortex. Some patients exhibited activations in areas associated with language function such as Broca’s area and the posterior part of the superior temporal gyrus (STG) regions, as well as cerebellum, posterior cingulate gyrus and superior frontal gyrus. Figure 3B shows the Harvard-Oxford probabilistic map of the pPHG (top), derived from structural data, used to define the pPHG ROI masks. The HTW paradigm consistently activated this region (red contour), with a maximum overlap of 84 patients (MNI152 Coordinates: x = −30, y = −41, z = −11) for the left pPHG ROI and a maximum overlap of 75 patients (MNI152 Coordinates: x = 30, y = −38, z = −12) for the right pPHG ROI.

### 3.1 Clinical versus FSL validation analysis

The following analysis of the reproducibility of the pPHG activation patterns is based on the 111 patients with at least one episode of repeated HTW-fMRI scans (7 patients had an additional episode, Figure 1). Reproducibility metrics were computed in 90 patients for which the validation analysis successfully revealed significant BOLD activations within the pPHG regions in both HTW fMRI sessions (see Section 3.2).

**Figure 4:**
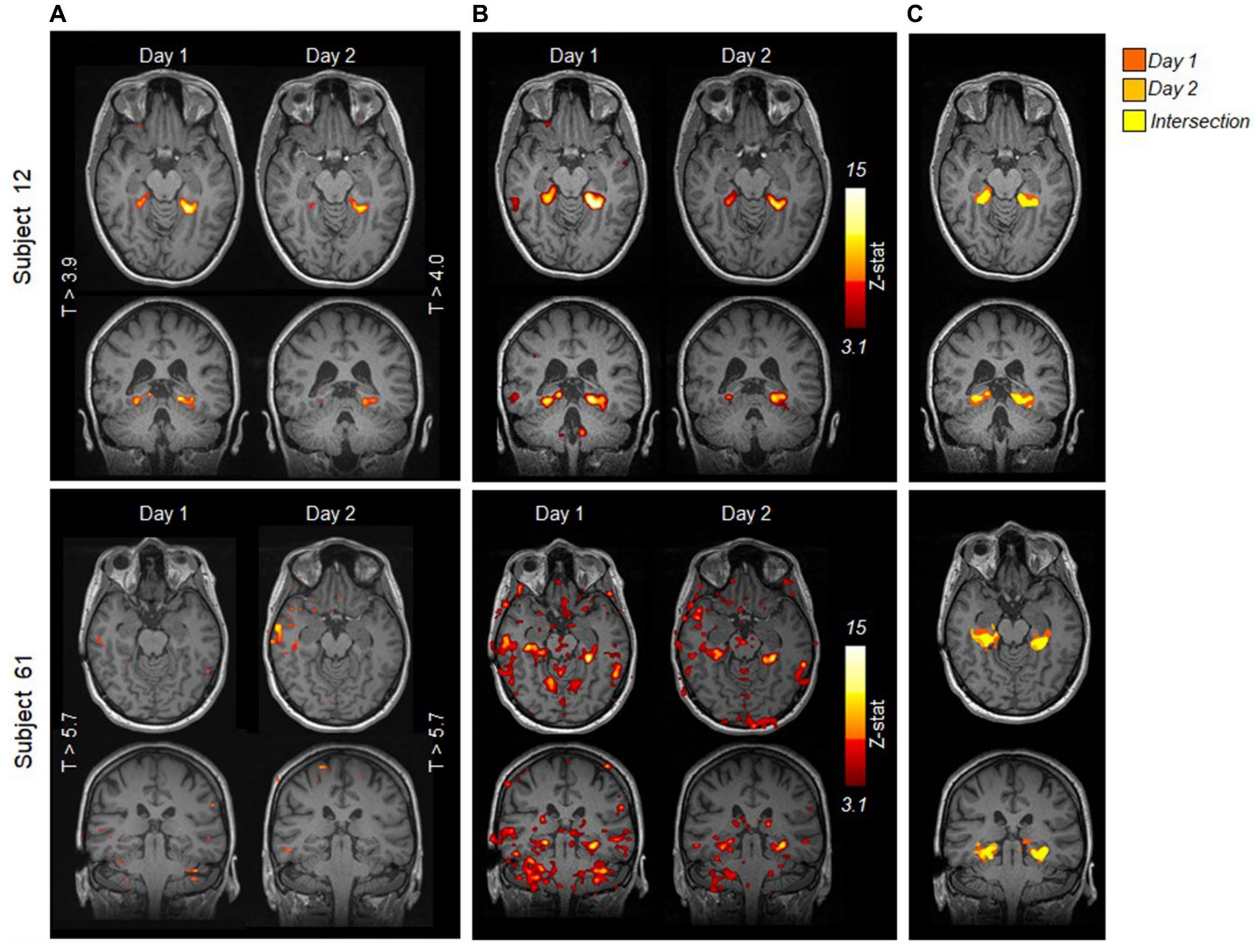
Comparison of statistical activation maps evoked by HTW task obtained with **(A)** clinical syngo.via analysis and **(B)** FSL validation analysis for two example episodes; a patient for whom the clinical analysis showed reproducible pPHG activity (top) and a patient for whom the clinical analysis fails to reveal any significant activation within pPHG in either day (bottom). Maps are overlaid onto T1-weighted anatomical MPRAGE from each day. **(C)** Comparison of FSL activation maps within pPHG probabilistic mask for each day on the patient’s reference anatomical volume. Color scale indicates activation from day 1 only (red), day 2 (orange) or both days (yellow).

A direct comparison of the results obtained with the clinical versus the FSL analysis is shown in Figure 4 for two example patients: for the patient on the top (subject 12), both analyses yield the same result, showing reproducibility of activations in the pPHG, whereas for the patient in the bottom row (subject 61), pPHG activations were reproduced with FSL validation analysis despite no activation revealed within either left or right pPHG in either of the days by the clinical analysis using syngo.via. Figure 4C shows the combined FSL maps across days in the same anatomical space masked by the probabilistic pPHG ROI. Activation patterns were deemed reproduced when there was overlapping activation across days (as shown by the yellow clusters) and the same lateralization was observed. If there were overlapping activations in one hemisphere but not in the other when either of the days showed bilateral activation (i.e., bilateral on one day and unilateral in the other), the activation patterns were considered to be partially reproduced.

The clinical analysis revealed that pPHG BOLD activations were observed in either left, right or both hemispheres in at least one of the days for 92 (78%) of the 118 episodes (87 patients). The validation FSL analysis showed an increased number of pPHG activations across patients, revealing pPHG activations for 108 (91.5%) of the episodes (102 patients). Note that both the clinical and FSL analysis revealed reproducible pPHG activation patterns on the first episode for 5 of the 7 patients with repeated episodes, while for the other two patients reproducible pPHG activation were only observed after the second episode.

**Figure 5:**
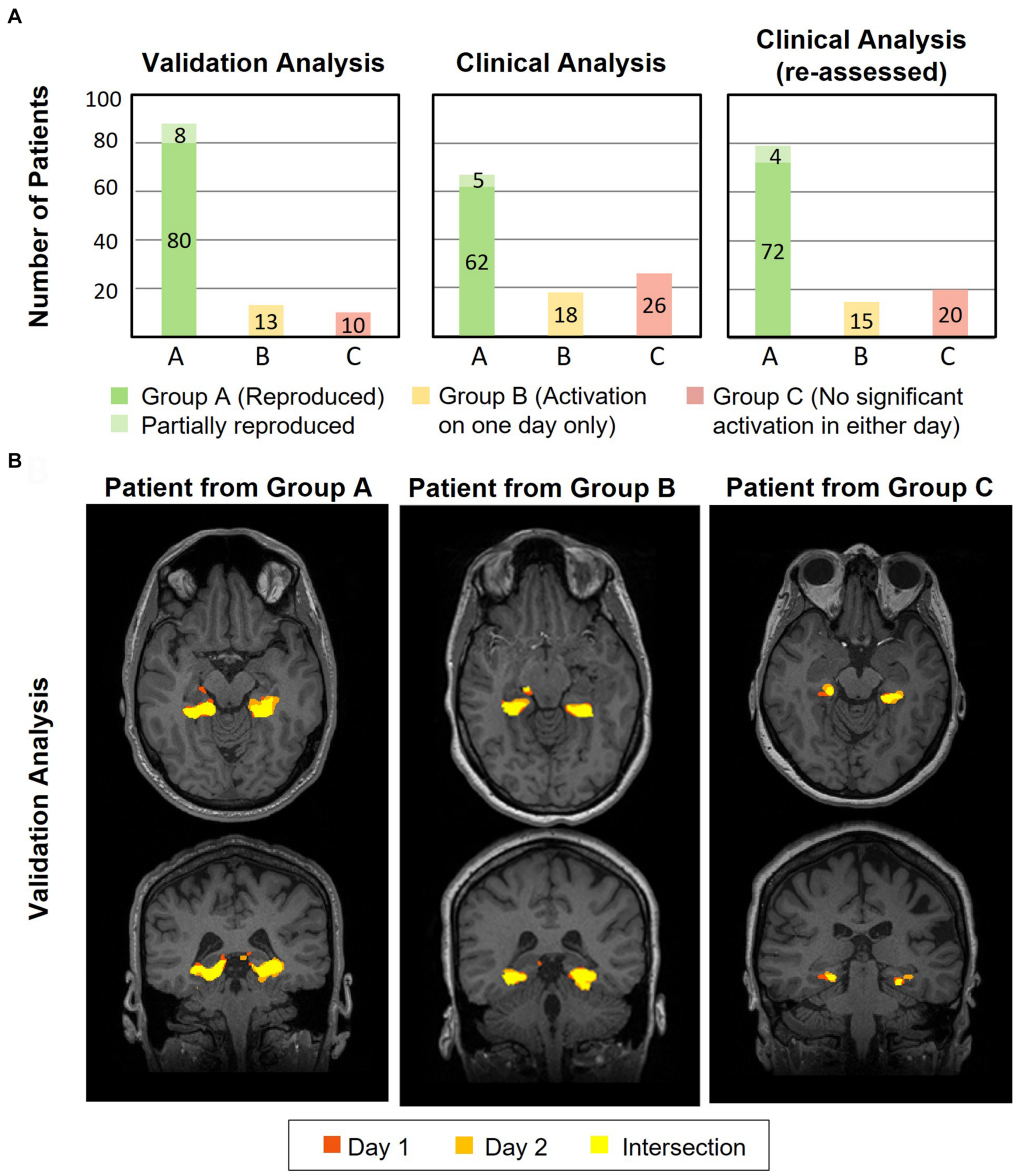
**(A)** Plots showing results for the validation (left) and clinical (middle and right) analysis of the number of patients for which the HTW paradigm evoked significant reproducible (overlapping) activations within the pPHG ROI across both days (green), patients with significant pPHG activation on one of the days only (yellow) and patients with no pPHG activation in either of the days (red) for those patients undergoing HTW fMRI in two sessions (considering first episode only). **(B)** Example of reproducibility of overlapping activation patterns within pPHG obtained with validation FSL analysis for patients from each of the clinical analysis groups; A–reproduced overlapping pPHG activation pattern, B–activation on one day only and C–no activation in either day.

Figure 5A compares the results from the FSL validation analysis (left) with the syngo.via clinical analysis (middle) across all 111 patients (excluding second episode for patients with repeated episodes). The FSL validation analysis revealed reproducible (overlapping) unilateral or bilateral pPHG activation patterns across days in 80 (72%) of the patients (green bar), (increasing to 82 (74%) after the second episode), while for a further 8 patients the pPHG activation patterns were deemed partially reproduced (light green), i.e., reproduced overlapping activations were found for the left pPHG but not for the right pPHG (left unilateral activation in one day and bilateral in the other). For the clinical analysis with syngo.via, the total number of patients showing reproducible pPHG activation patterns after the first episode was 62, (64 after second episode), with 5 further patients showing unilateral pPHG activation in one day and bilateral pPHG activation patterns in the other (partially reproduced). It is worth noting that in some patients where only unilateral pPHG activations were observed with the clinical analysis, the FSL analysis revealed bilateral activations (although this additional cluster was not always observed across both days). The FSL validation analyses generated reproducible activations for all patients whose activation had been deemed clinically reproducible, except for two patients whose activation was deemed partially reproduced due to the lack of overlap of right pPHG activation volumes across days. For the clinical analysis, 18 patients showed significant activations within pPHG regions in one of the days but not the other (yellow bar) and 26 further patients did not show significant activation within the pPHG region in either day (red), with this number reducing to 24 after the second episode. For the FSL analysis, only 10 of the patients (9 after the second episode) did not show any activation in either day. This number includes patients who had failed to prepare, were not able to perform the task, exhibited excessive motion or in one case abandoned due to a headache.

The improved reproducibility of FSL is shown in Figure 5B for 3 example patients (one from each group) with varied results on clinical analysis. FSL produced reproducible pPHG activations for some of the patients with no reproducible activation pattern when analyzed with syngo.via. We note that in some cases the statistical threshold used in the clinical analysis was significantly higher for patients with no reproducible activation (e.g., t > 5.7 for patient 61, versus t > 3.9 and 4.0 for patient 12 in Figure 4A). A re-evaluation of the clinical analysis in these patients revealed reproducible activations within pPHG in 10 further patients when lowering the t-stat threshold or simply by removing the clustering (Figure 5A, right).

### 3.2 Reproducibility metrics

Results of the pPHG activation volumes for both days as well as R_size_ and R_overlap_ metrics were compared across scanners first (Table 2). Note that two of the patients were scanned in both scanners (on the Verio and Skyra for the first and second episode respectively), hence yielding a total of 113 patients of whom 58 were scanned on scanner 1 and 55 on scanner 2. For patients who had two episodes on the same scanner (*n* = 3, scanner 1; *n* = 2, scanner 2), only the second episode was used. T-tests were computed for the activation volumes for each individual day and each reproducibility metric on left and right pPHG ROIs independently to test whether the means across scanners were significantly different. F-tests were first computed to determine what type of t-test should be performed (i.e., unequal or equal variance t-tests). There was a trend for larger activation volumes of the pPHG regions on Scanner 2 compared to Scanner 1, although this was only marginally significant (*p*-value = 0.036) for the Left pPHG ROI on day 1. Despite this trend for larger activation volumes on Scanner 2, results for the R_size_ and R_overlap_ metrics were not significantly different across scanners.

**Table 2:** Summary of patient details and results across scanners.

	Scanner 1	Scanner 2
Patient details		
Number	58	55
Sex (Male/Female)	28/30	30/25
Age (mean ± std., [range])	38 ± 12 yr., [18,65]	36 ± 11 yr., [18,60]
Activation volume (mL)		
Left pPHG (day 1)	1.7 ± 1.2	2.2 ± 1.8
Left pPHG (day 2)	1.8 ± 1.3	2.5 ± 2.3
Right pPHG (day 1)	1.4 ± 1.2	1.9 ± 1.5
Right pPHG (day 2)	1.5 ± 1.1	1.8 ± 1.7
R_size_		
Left pPHG	0.73 ± 0.21	0.68 ± 0.31
Right pPHG	0.68 ± 0.33	0.65 ± 0.34
R_overlap_		
Left pPHG	0.52 ± 0.21	0.51 ± 0.25
Right pPHG	0.40 ± 0.25	0.43 ± 0.28

**Figure 6:**
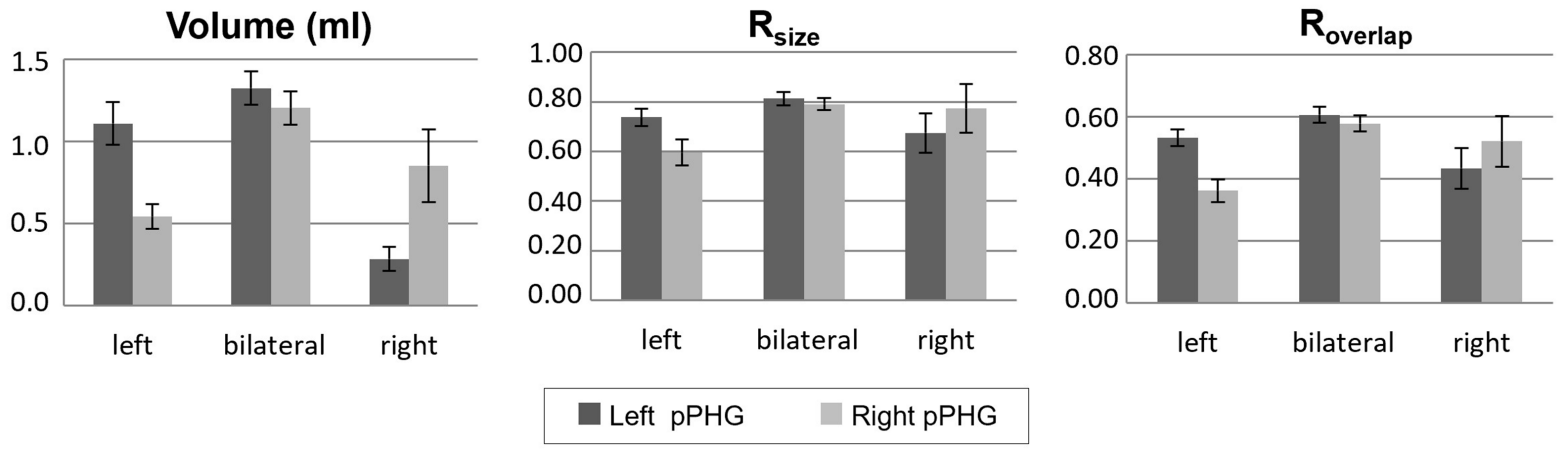
Volume of activation (averaged across days) and reproducibility metrics; R_size_ and R_overlap_, within the left and right hemisphere pPHG ROIs for each group of patients (mean across patients; error bars: standard error across subjects). Patients grouped according to their lateralization index (averaged across days).

Hence, data from both scanners were grouped together. Patients who showed activations within the pPHG (left and/or right hemisphere) in both sessions based on the FSL analysis, including patients for whom activation was unilateral in one day and bilateral in the other (90 patients in total) were included in the following analysis to assess reproducibility of lateralization. Results were grouped according to each patient’s average lateralization index across days; comprising 43 patients with left lateralization, 9 patients with right lateralization and 38 patients with bilateral pPHG activations. Figure 6 shows the mean activation volume (mL, mean across both days), R_size_ and R_overlap_ metrics across patient groups for left and right pPHG ROIs. The R_size_ metric shows that at least 74% of the volume of activation was reproduced for the dominant hemisphere, with 81 and 78% of the volume reproduced on average for left and right ROIs, respectively, in patients with bilateral activations. The R_overlap_ metric reflects the level of overlap between activation volumes across sessions. For patients with strong unilateral activations, the mean overlap of the activation volumes for the dominant hemisphere was at least 52%, whereas for patients with bilateral pPHG activations there was a mean 60% and 57% volume overlap for left and right pPHG activations across days, respectively.

**Figure 7:**
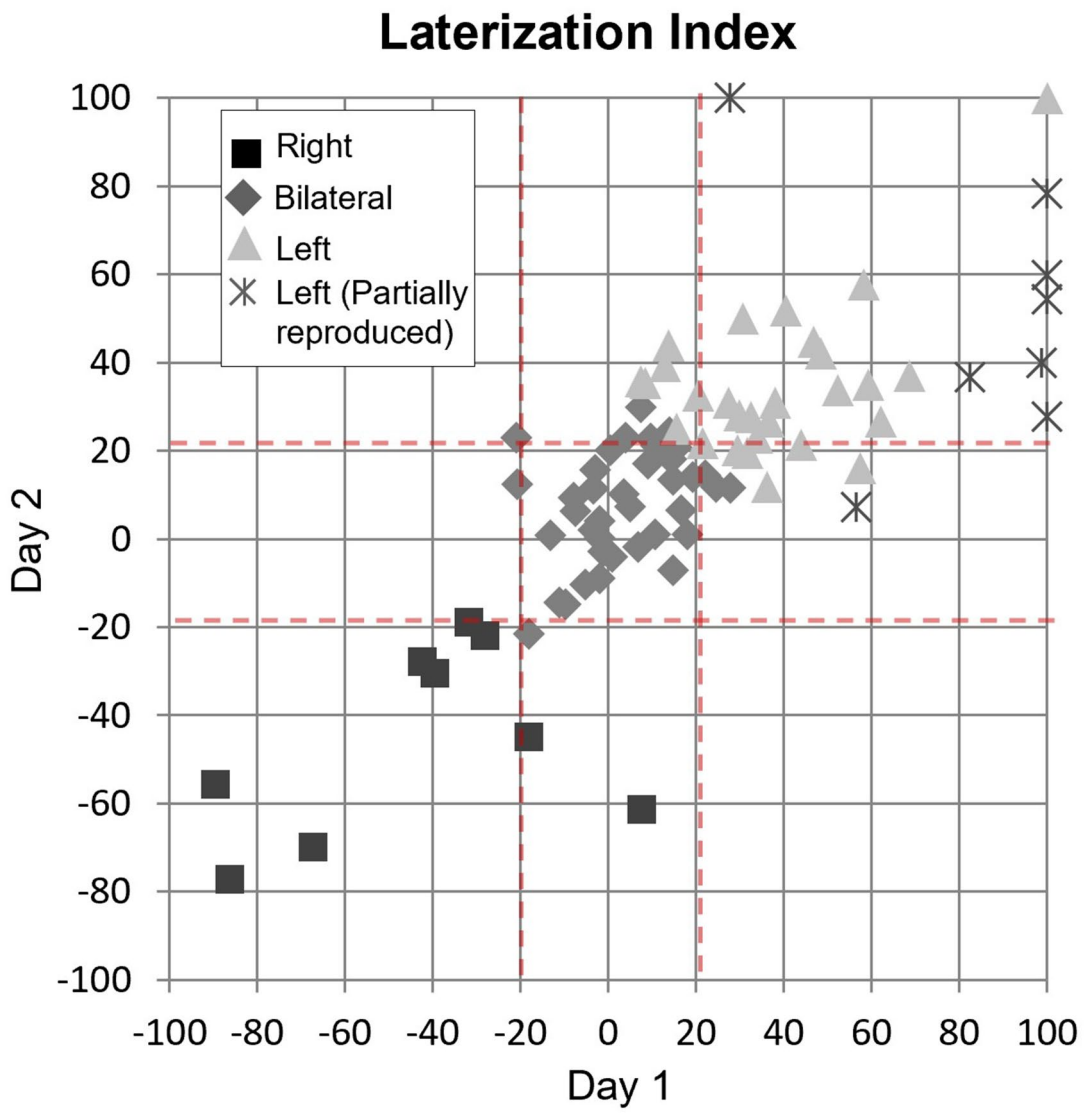
Correlation of lateralization indices between days shown for each group of patient’s patterns (square: right lateralization; diamond: bilateral; triangle: left lateralization) with reproduced activation and for those with partial (left reproduced but not right) activation pattern (cross). Dash red lines: threshold used to determine lateralization (LI > 20 left, LI < −20: right, −20 < LI <20, bilateral). The Pearson correlation coefficient for these data was 0.78.

The correlation analysis of the pPHG Lateralization Index (LI) showed a high level of correlation (0.78, Pearson’s coefficient) between LIs across days. Figure 7 plots for each patient the LI generated from day 2 pPHG activation volumes against corresponding LI obtained from day 1. Patients are color-coded according to their average lateralization index: 39 (43%) patients had an average LI between −20 and 20 and were classified as bilateral; 9 (10%) patients had an average LI smaller than −20 and hence predominantly right activation, while the remaining 42 (47%) patients show predominantly left activation (LI > 20), including the patients who showed partially reproduced activation (represented by crosses). Note that six of these patients did not have reproducible (overlapping) activations within the right pPHG due to lack of any significant activation within this area in one of the days (hence LI = 100), for the remaining two patients there was no overlap due to small/weak right pPHG activation on one of the days. However, for the day with bilateral activations, the activation was predominantly left according to the LI classification (range 28 to 78) in all patients but one for whom LI = 7. The Intra-class correlation coefficient obtained based on a 2-way ANOVA for these data was 0.76, indicating high reproducibility (low inter-session variability compared to the inter-patient variability).

When examining whether the lateralization classification according to the LI metric was in agreement with the subjective clinical report (for the 86 patients showing activation in at least one day), we found that there was agreement for a total of 63 patients (including 49 patients with reproducible activation patterns across days and 14 patients when there was only significant activation in one day) as well as for one of the days in six patients with partial reproducible activations. Of the sessions for which the lateralization classification based on LI disagrees with the clinical report, we found 17 cases with LI of 34 ± 14 (mean ± std., range 22 to 78) where the clinical report has stated ‘bilateral symmetrical activation’ or ‘bilateral activation’ (without left or right predominance); 3 cases (LI = 11 ± 6, range 4 to 15) where the clinical report stated ‘left’ or ‘predominantly left’ activation, and 3 cases (LI = −11 ± 6, range − 15 to −3) where the clinical report stated ‘right’ or ‘predominantly right’ activation.

## 4 Discussion

The aim of this study is to assess the reproducibility of the HTW paradigm for visuospatial memory mapping at the single-subject level to demonstrate its utility for translational clinical application. Preoperative fMRI using the HTW paradigm is useful for identifying patients at high risk for visual memory decline based on the asymmetry of hemispheric activations. Janszky et al. (4) showed that all patients showing larger activation to this task on the side later operated on compared with the contralateral side had a postoperative memory decline.

We found that most patients were able to perform the HTW memory task successfully with adequate preparation. Only a few negative studies were observed (9% of patients in either day, 11% of patients in one of the days), and those typically lacked activation anywhere else in the brain, which could be attributed to factors such as the inability to perform the task, excessive motion artifacts or sub-optimal technical quality of the EPI data. In addition, one patient abandoned the scan session due to a headache. We found that with education, training and repetition of the task, significant activations were successfully obtained within pPHG regions in most patients (79% of patients in the first episode and 81% of the patients after repeating the episode). The Pearson correlation and ICC results of 0.78 and 0.76, respectively, indicate high reproducibility of the LI metric across sessions. Given that asymmetry of hemispheric activations has been shown to correlate with post-operative memory outcome in previous studies (4, 10, 11, 40), the reliability of this metric is essential to demonstrate the clinical usefulness of this paradigm. Our results suggest that the HTW paradigm is doable by patients and appropriate for clinical scans. Crucially, the HTW paradigm produced similar results across the two scanners employed despite substantial differences in hardware specifications, software platforms and acquisition protocols (Table 2). The robustness of the HTW paradigm to changes in scanner type and acquisition parameters is necessary to demonstrate its clinical utility.

### 4.1 Clinical analysis versus FSL validation analysis

Activations within the pPHG regions were successfully observed in 62% of patients using subjective clinical analysis but this increased to 81% using automated FSL analysis in individual epilepsy patients. The FSL analysis not only validated the subjective clinical results but also offered increased sensitivity and reliability. Some of the patients with reproducible pPHG activations with FSL but negative clinical results had strong activations elsewhere in the brain. In these cases, a higher statistical threshold had been used in an attempt to confidently identify memory specific activations, obscuring the activations present in the pPHG region in the process. After the clinical analysis was retrospectively re-assessed by altering the cluster size and/or adjusting the statistical threshold, the number of patients with positive reproduced pPHG activations increased to 70%. However, there was a group of 8 patients with typically small FSL activation volumes for which reliable pPHG activations could not be confidently observed using subjective clinical analysis even when lowering the statistical threshold. The difference in results between the analysis methods may arise due to differences in the pre-processing steps, such as different motion correction or smoothing algorithms used by syngo.via and FSL. The smoothing algorithm (SUSAN) employed by FSL averages a voxel only with local voxels which have similar intensity as opposed to standard Gaussian filters which average across all neighboring voxels, hence reducing noise while preserving the small structures. Another advantage of the validation analysis is that activations for both days were directly compared in the same anatomical space, in contrast to the clinical analysis where activations are assessed in the anatomical space acquired in the same session. By using the same anatomical space, it was possible to assess whether small volumes of activations across days overlap, distinguishing these from noise, as well as confirm that the activations were indeed in the expected region by using the probabilistic pPHG mask.

### 4.2 Reproducibility of pPHG activations

Despite the need for reliable memory paradigms in the clinical setting, previous studies assessing the reproducibility of fMRI results generally report poor reproducibility of brain activations, as discussed in the introduction with lower reproducibility reported in patients than in healthy controls (41). However, some studies have shown better reliability where memory encoding tasks are used rather than retrieval (42, 43). The HTW paradigm, which is a visuospatial retrieval task, has previously shown superior reproducibility of activations within the MTL compared to other memory tasks in a small number of epilepsy patients (29).

Test–retest reliability of fMRI results can also be inferred by investigating the volume ratio that measures reproducibility in the number of voxels (activation volume size) and the overlap ratio that measures location of activation by comparing voxels activated in both sessions to those activated in only one session. The reproducibility of the activation volume in our study was on average 74/77% for the dominant pPHG hemisphere in patients with left/right lateralization, and 81/78% for left/right pPHG ROIs for patients with bilateral activations. This is higher than the average reproducibility of 63% previously reported for the bilateral parahippocampal region in a study using a memory encoding task of complex visual pictures (44). It is also higher than the relative volume ratios reported in a study (28) comparing reproducibility of three different memory encoding tasks (20–70%).

The volume of overlap metrics obtained in the current study (R_overlap_ range 0.52–0.60) also indicate that the HTW paradigm shows superior reproducibility of the location of activation than previous memory encoding studies, which report R_overlap_ values in the range 0.18–0.4 depending on the paradigm used (44, 45). Harrington et al. (28) reported a higher mean R_overlap_ of approximately 0.55 using a scene encoding memory paradigm, but with very high variability between subjects in a small cohort of 18 healthy volunteers. The high test–retest reproducibility across the large cohort of TLE patients in the current study therefore demonstrates the value of HTW as a reliable paradigm for memory activation in a clinical setting.

The Intra-Class Correlation Coefficient (ICC), which represents the ratio of between subject variance to the total variance, can also be used as a metric to assess reproducibility of brain activations across sessions in specific ROIs. ICC is higher when variance across sessions is low; Bennett and Miller (46) suggested a range of ICC values between 0.33 and 0.66 within which fMRI studies are typically reliable, while Fleiss (47) proposed a classification whereby ICC values below 0.4 indicate ‘poor’ reproducibility, values between 0.4 and 0.59 indicate ‘fair’ reproducibility, values from 0.6 to 0.74 represent ‘good’ reproducibility and values above 0.75 indicate ‘excellent’ reproducibility. According to this classification, the 0.76 ICC value obtained across 90 patients for memory LI in the present study indicates excellent between session stability. This represents higher stability compared with previous studies using ICC to assess reproducibility of memory LI; showing a lack of stability (ICC = −0.71) for memory encoding LIs, but moderate stability (ICC = 0.45) for memory retrieval LIs across sessions (24), and good stability (ICC = 0.65) for verbal memory LIs but low stability (ICC = 0.35) for visual memory (25). Interestingly, Buck et al. (24) also showed that while memory encoding LIs were not stable across sessions, LIs for language were stable across repeated sessions, highlighting the challenge that memory fMRI poses in comparison to language mapping. However, the ICC obtained for language LI in that study (ICC = 0.71) was inferior to our ICC result obtained for visuospatial memory retrieval LIs with the HTW task. Some studies that showed good ICC reliability at the group level did not find reproducibility across individuals, particularly for memory encoding (26) showing poor reliability (ICC < 0.3) for the medial temporal region across three encoding paradigms (word, scene, fractal). In the study by Brandt et al. (26) two of three paradigms (words paradigm, fractals paradigm) yielded poor reproducibility of brain activation also for the overall activation network, suggesting that the low reproducibility of brain activation is not limited to regions prone to susceptibility artifacts (such as the hippocampus), but may be more dependent on the paradigm. A study investigating reproducibility of a verbal memory task across MCI patients and controls (27) found no significant reproducibility in controls but significant ICC (0.48) in patients for the hippocampus bilaterally during the retrieval but not during memory encoding condition.

### 4.3 Home Town Walk paradigm

The Home Town Walk paradigm appears effective in eliciting activation within the posterior PHG in most patients being evaluated for temporal lobe epilepsy. It has been suggested that encoding tasks yield increased BOLD signals in the posterior medial temporal region while retrieval tasks yield activations in the anterior medial-temporal region (48). While Janszky et al. (4) and Towgood et al. (29) did not specify the exact location of memory specific activations within the temporal lobe evoked by the HTW task, a recent study using a modified version of the HTW task used a mask of the posterior part of the hippocampus and parahippocampal gyrus for their analysis (49). A study comparing activation patterns between the HTW task and a picture encoding task (50) specifically described group activation maps with a large overlap within the PHG region, with the HTW task yielding stronger activation in the anterior portion of the PHG compared with the encoding task. Given this segregation of anterior and posterior PHG regions for memory retrieval and encoding, it has been hypothesized (25) that an fMRI protocol that maps both the encoding and retrieval phases of the memory process could yield extended BOLD activations and improve the reliability of memory mapping at the individual patient level, compared to other paradigms that only involve the encoding or retrieval phase. The HTW paradigm involves retrieval of deeply encoded long term memory induced by self-paced performance of an imaginary walk. Although we did not scan patients during the encoding phase, we found that a good preparation of this phase was crucial to obtained robust activations within the medial temporal lobe. Our probabilistic maps of the HTW activation patterns generated across 114 patients largely overlap with the posterior PHG ROI defined from the Harvard-Oxford probabilistic atlas of this region (Figure 3B), a region that has been previously activated by memory encoding tasks [e.g., (50)].

The parahippocampus receives convergent input from the various cortical sensory association areas and provides most of the cortical input to the hippocampal formation. Numerous memory encoding studies have focused on the role of the anterior hippocampus pre and post surgically, finding that the greater the activation within the anterior temporal lobe, the greater the verbal and visual memory decline after left and right ATLR, respectively [e.g., (2, 11, 51)], and it has also been shown that greater activation in the posterior part of the ipsilateral hippocampus preoperatively correlated with better verbal or visual memory outcome post-operatively (52). However, there is evidence that activation in other regions beyond the hippocampus, including the parahippocampus, can also predict visual memory outcome following ATLR (4, 40); Interestingly, Rabin et al. (40) showed that activation asymmetry to a visual encoding task in the MTL ROI comprising the hippocampus, parahippocampus, and fusiform gyrus correlated significantly with memory outcome and WT laterality, whereas for the hippocampus ROI only absolute activation correlated significantly with memory outcome.

Despite the HTW paradigm having been previously described as a visuospatial with non-verbal memory components, we found that some patients show activations of language specific processing areas such as Broca’s area and the posterior part of the superior temporal gyrus (STG) in the dominant hemisphere (see Figure 3A). Hence this paradigm reflects verbal components to a certain extent. We requested that patients participating in this study prepare their home town walk in 10 steps recalling as many details as possible and writing them down. These details of the mental navigation may include other stimuli beyond visual landmarks, such as auditory stimuli (i.e., a barking dog) and the smell of the surroundings, as well as language stimuli (i.e., reading road signs). The involvement of verbal navigation strategies may explain why some patients exhibited activations in language specific areas.

Although we cannot control for the level of verbal navigation strategies used by each patient, the HTW paradigm is a practical and conceptually simple task (compared with other memory tasks) and we believe that most medical centers should be able to conduct this test in a standardized way. Our results have shown that patients are able to perform this task reliably and are motivated to do the necessary preparation given appropriate instructions. Previous studies employing the HTW task (4, 10) have also emphasized the suitability of this paradigm for patients with epilepsy and cognitive impairment. Furthermore, a recent study showed a modified version of the HTW paradigm involving mental navigation associated with activities at home and school was effective in activating MTL structures in pediatric epilepsy patients (49).

### 4.4 Limitations

Limitations of this study include the use of single institution data and the absence of healthy controls. However, these constraints are consistent with the clinical environment in which fMRI is likely to be used. We have not investigated factors that may affect the reproducibility of memory fMRI results, including differences in patient’s cognitive ability, potential memory plasticity, the duration and severity of epilepsy, the time elapsed from the patient’s last seizure to the MRI scan, as well as the effects of medication or other treatments. Future work will need to assess whether reproducibility of the results depends on memory impairment or specific disease characteristics.

Another limitation of this study is that the HTW paradigm is primarily a visuospatial memory retrieval task which may be used for prognosticating post-surgical visual memory decline as per AAN guidelines, but not for predicting postoperative verbal memory changes. In order to comprehensively evaluate both visual and verbal memory functions and postsurgical outcomes, an additional verbal memory paradigm such as word list learning or story recall, should be used in conjunction with HTW. Alternatively, language encoding fMRI paradigms could be considered for predicting verbal memory outcome, as suggested by the AAN guidelines (12). A study by Binder et al. (14), showed that lateralization of language correlated with lateralization of verbal memory and found that preoperative fMRI was more useful than Wada memory testing to identify patients at risk of verbal memory lost following left anterior temporal lobe resection. In our practice, we use a combination of a visual–spatial memory paradigm, language paradigms and neuropsychological cognitive assessment for predicting post-surgical memory deficits. This comprehensive approach is aimed at accurately predicting both visual and verbal memory declines post-surgery. Future work in those patients proceeding to surgery will assess how well HTW and language paradigms activation patterns could effectively predict post-surgery visual and verbal memory outcomes respectively, and whether there is a risk stratification in relation to whether activation is symmetrically bilateral or ipsilateral to the region intended for resection.

## 5 Conclusion

Reliable and robust memory fMRI data has been obtained at the single-subject level in a clinical setting. The findings demonstrate the clinical utility of the HTW fMRI paradigm showing highly reproducible detection and lateralization of visuospatial memory activation in individuals with TLE. The Home Town Walk paradigm should be used alongside language fMRI and other verbal memory tasks, such as word list learning, and story recall, as well as neuropsychological clinical assessments. This comprehensive approach ensures a thorough evaluation of memory function. Investigation of the value of HTW fMRI for predicting post-surgical memory function in TLE patients and its potential clinical impact is warranted.

## Data availability statement

The raw data supporting the conclusions of this article will be made available by the authors, without undue reservation.

## Ethics statement

Ethical review and approval was not required for the study on human participants in accordance with the local legislation and institutional requirements. Written informed consent from the patients/participants or patients/participants’ legal guardian/next of kin was not required to participate in this study in accordance with the national legislation and the institutional requirements. Written informed consent for the publication of anonymized imaging data acquired for clinical purposes was provided by the relevant participants.

## Author contributions

RSP: Data curation, Formal analysis, Investigation, Methodology, Project administration, Software, Validation, Visualization, Writing – original draft. RF: Data curation, Formal analysis, Methodology, Writing – review & editing, Investigation, Validation. RW: Data curation, Formal analysis, Methodology, Writing – review & editing, Validation. RJ: Data curation, Formal analysis, Methodology, Writing – review & editing, Validation. JH: Investigation, Methodology, Writing – review & editing. SS: Supervision, Writing – review & editing, Conceptualization. AB: Supervision, Writing – review & editing. RC: Supervision, Writing – review & editing, Conceptualization. ND: Conceptualization, Data curation, Formal analysis, Investigation, Methodology, Project administration, Supervision, Writing – review & editing, Validation. VS: Conceptualization, Data curation, Formal analysis, Investigation, Methodology, Project administration, Supervision, Validation, Writing – original draft.

## Footnotes

^1^ https://identifiers.org/neurovault.collection:262
