# Supplementary material for: Test-retest reliability of the “Home Town Walk” fMRI paradigm for memory activation and lateralization in the pre-surgical evaluation of patients with temporal lobe epilepsy
Source: Front Neurol. 2024 Jul 23;15:1419047. doi: 10.3389/fneur.2024.1419047 (PMC11300360; doi:10.3389/fneur.2024.1419047)
Supplement: Supplementary file 1 [file Presentation_1.pdf]

Functional MRI (fMRI) Examination
Memory Walk

Preparation for this scan is critical. If you do not come prepared, we may not be able to perform your scan.
Please ring us if you have difficulties preparing or you require the information in a different format.

This examination is designed to visualise the exact area of your brain which is responsible for your memory.

This information sheet is designed to prepare you for the examination and explain what we require you to do prior to attending for the scan.

Coming prepared for the scan is extremely important and is crucial to the studies success. You will be asked to attend on two days which will be very close together and you must have prepared for the scan on Day 1, having filled in the crib-sheet provided.

We need you to have a short walking journey (about 10 minutes) that you know well, planned in your head, for example, a walk around your local park, or to the local shops. Try and split the journey up into 30 second sections. There is an example over-leaf to demonstrate which shows a walking journey to work. The journey/things you remember must be real.

This part of the examination will take about 10 minutes of continuous scanning. We will ask you to recall your journey or experience in a methodical way and in as much detail as possible. During your scan you will be able to see a screen which will tell you when to ‘Walk’ and ‘Count’.

During the ‘Walk’ periods, recount your walk in as much detail as possible. During the ‘Count’ periods, silently count down from 100. The ‘Walk’/’Count’ periods will alternate for 10 minutes until the scan is complete. If you get to the end of your walk, go back to the beginning and start again. If you lose your place in your walk during the scan, go back to the beginning and start again. If you don’t get to the end of your walk, it does not matter.

Plan your 10 minute journey into the 10 methodical blocks and try to have at least five points for each block.

The crib sheet provided is for you to make notes. Whilst this will not be available during the scan, it is important to fill this out in as much detail as possible and practice your journey before you come in for the scan.

This is not a test of your memory, and we will not know what you are thinking.
For the scan to work, it is important that you are accessing real memories in your mind.

It is important whist performing this task that you do it entirely in your head; do not speak the steps out loud. It is also important that you keep your body absolutely still as movement can affect the quality of the scan.

The radiographers will go through these instructions before you start the examination and again before each scan so you can ask about anything you are unsure of.

Below are some examples of the images we will produce, with the bright orange areas being the areas of the brain that are being used.

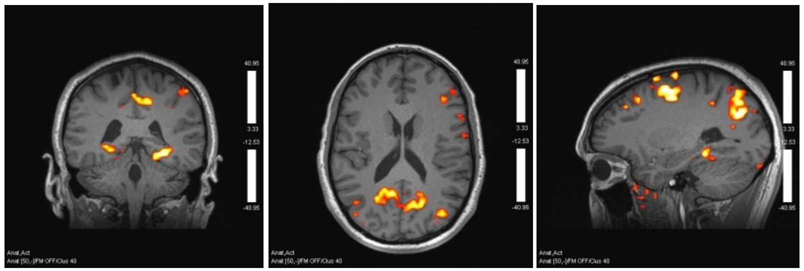

If you have any questions or concerns, please contact:

Jane Herbert – Band 7 MRI Radiographer 0121 371 2233
Eleanor Derrick – Band 7 MRI Radiographer 0121 371 8035

**Document Name:** Memory walk	**Author:** JH/ED	**Issue Date:** .1.2.2021
**Version:** 2	**Authorised By:**	**Review Date:** 2021
